# Macrophage Polarization in Obesity and Type 2 Diabetes: Weighing Down Our Understanding of Macrophage Function?

**DOI:** 10.3389/fimmu.2014.00470

**Published:** 2014-09-26

**Authors:** Michael James Kraakman, Andrew James Murphy, Karin Jandeleit-Dahm, Hélène L. Kammoun

**Affiliations:** ^1^Cellular and Molecular Metabolism Laboratory, Baker IDI Heart and Diabetes Institute, Melbourne, VIC, Australia; ^2^Haematopoiesis and Leukocyte Biology Laboratory, Baker IDI Heart and Diabetes Institute, Melbourne, VIC, Australia; ^3^Department of Immunology, Monash University, Melbourne, VIC, Australia; ^4^Diabetic Complications Laboratory, Baker IDI Heart and Diabetes Institute, Melbourne, VIC, Australia; ^5^Department of Medicine, Central Clinical School, Monash University, Melbourne, VIC, Australia

**Keywords:** macrophage, inflammation, obesity, type 2 diabetes, M1/M2, polarization

## Abstract

Obesity and type 2 diabetes are now recognized as chronic pro-inflammatory diseases. In the last decade, the role of the macrophage in particular has become increasingly implicated in their pathogenesis. Abundant literature now establishes that monocytes get recruited to peripheral tissues (i.e., pancreas, liver, and adipose tissue) to become resident macrophages and contribute to local inflammation, development of insulin resistance, or even pancreatic dysfunction. Furthermore, an accumulation of evidence has established an important role for macrophage polarization in the development of metabolic diseases. The general view in obesity is that there is an imbalance in the ratio of M1/M2 macrophages, with M1 “pro-inflammatory” macrophages being enhanced compared with M2 “anti-inflammatory” macrophages being down-regulated, leading to chronic inflammation and the propagation of metabolic dysfunction. However, there is emerging evidence revealing a more complex scenario with the spectrum of macrophage states exceeding well beyond the M1/M2 binary classification and confused further by human and animal models exhibiting different macrophage profiles. In this review, we will discuss the recent findings regarding macrophage polarization in obesity and type 2 diabetes.

Inflammation is a fundamental biological process whose role is not only to enable host protection against pathogens, but also to stimulate and modulate repair and healing when cellular damage occurs. During an inflammatory event, once the initial insult is contained, a primary objective is the restoration of tissue homeostasis. Failure to appropriately resolve an inflammatory stimulus can result in persistent immune system activation, which can actually cause tissue damage and disease. Significant literature over the last decades has established obesity to induce a state of chronic low-grade systemic inflammation (1). Importantly, the inflammation accompanying obesity is distinctly different to that of acute inflammation, as the inflammatory stimulus fails to be resolved. This is of particular significance as chronic low-grade inflammation is implicated in the etiology of atherosclerosis, hypertension, type 2 diabetes (T2D), and even certain cancers, which can all be associated with obesity (2, 3). Considering the economic burden from the increasing prevalence of these chronic metabolic diseases, it is not surprising that considerable scientific attention has focused on how and why obesity promotes chronic low-grade inflammation.

Immune cells are the primary effectors of most inflammatory reactions and are categorized into the innate and acquired immune systems. With respect to obesity, leukocytes from both immune systems have been implicated in the development of chronic low-grade inflammation and metabolic dysfunction (1). However, adipose tissue macrophages have received the lion’s share of attention of the immune cells involved. Macrophages can display remarkable phenotypic heterogeneity with the ability to perform vastly different roles depending on the biological situation (4). Thus, a spectrum of many different macrophage populations has been characterized by combinations of membrane markers and gene expression profiles. This led to the establishment of a complex nomenclature, which over time has become simplified into two main macrophage phenotypes, M1 pro-inflammatory and M2 anti-inflammatory macrophages. This classification is obviously a simplistic view of the situation as it is now clear that macrophages dynamically evolve from one phenotype to the other according to their environment and can occupy various points of the spectrum with mixed characteristics [for updated nomenclature see Ref. (5, 6)]. For clarity purposes, we will mostly mention macrophages as either M1 or M2.

## Macrophage Accumulation in the Obese Adipose Tissue

Phagocytosis is a main function of macrophages that allows them to contribute to tissue homeostasis through surveillance, maintenance, and repair. Though long-lived macrophage populations reside within almost all bodily tissues, in 2003, two separate laboratories reported macrophage accumulation in the white adipose tissue (WAT) of both obese patients and rodents (7, 8). Importantly, this was completely in line with Hotamisligil’s landmark article 10 years earlier observing TNFα secretion from the adipose tissue in obese rodents (9). Indeed, it appeared these macrophages were the source of the elevated inflammatory cytokines reported in obesity and their accumulation was associated with insulin resistance (7, 8). These landmark studies were the first to link obesity and insulin resistance with adipose tissue macrophage content and inflammation. Later it appeared that the accumulation of macrophages is not limited to the WAT in obesity with macrophages found to accumulate in many other organs critical for glucose homeostasis such as liver, pancreas, gut, and even the brain (3). Regardless, the most significant immunological changes occurring during obesity originate within the adipose tissue. Of interest, despite the adipose tissue representing a small portion of whole body glucose disposal, immune dysfunction within this tissue is sufficient to impair systemic glucose metabolism (1).

## Macrophage Polarization in Obesity and T2D

Macrophages constitute an important fraction of non-adipocyte cells within the WAT. Indeed, within a “normal” lean WAT, they can account for almost 10% of total cell number. Profiling of lean adipose tissue revealed these macrophages to possess an M2 phenotype. These macrophages perform tissue surveillance and remodeling functions and are associated with maintaining WAT insulin sensitivity. Indeed, the manipulation of peroxisome proliferator activator receptor (PPAR) transcription factors required for macrophage M2 polarization was associated with metabolic dysfunction (10–12). The current theory supports that weight gain induces local inflammation and chemokine production to promote recruitment of circulating pro-inflammatory (Ly6C^hi^) monocytes. Recruited monocytes differentiate into an M1 macrophage phenotype and their accumulation leads to an imbalance between M1 and M2 macrophages. Increased cytokine production from M1 macrophages and/or reduced anti-inflammatory signals from the M2 macrophages promote adipose tissue dysfunction and impairs glucose tolerance.

Evidence for the detrimental role of M1 macrophages in promoting adipose tissue insulin resistance has been reported in several studies. Lumeng and colleagues first reported that the macrophages accumulating in obese WAT possessed an inflammatory CD11c^+^ M1 phenotype and gathered around necrotic adipocytes in Crown like structures (CLS) (13, 14). Furthermore, M1 macrophage numbers were shown to correlate with insulin resistance in high-fat fed rodents (15). Whether WAT M1 macrophages could be targeted therapeutically has proven more challenging. Clodronate is a toxic compound administered in liposomes that gets taken up by macrophages thereby inducing their apoptosis. Clodronate liposome injections succeed in decreasing visceral WAT macrophage accumulation and improve glucose metabolism and insulin sensitivity in HFD-fed mice (16). However, this approach has limitations as clodronate liposomes targeted all WAT macrophage phenotypes as well as liver Kuppfer cells. One could envisage a deleterious effect from depleting M2 macrophages on a long term basis. A more specific removal of M1 macrophages was achieved in an elegant study published by Olefsky’s group. They demonstrated that ablation of M1 macrophages, achieved by targeting diphtheria-sensitive CD11c^+^ cells with diphtheria toxin, was associated with improved glucose tolerance (17). While CD11c expression allows discrimination between M1 and M2 macrophages, its expression is not exclusive for M1 macrophages. Dendritic cells and neutrophils also express CD11c and the elevation of these cells in obese WAT may contribute to the increased CD11c^+^ cell population and insulin resistance (18, 19). From these studies, it is clear that targeting established M1 populations within obese WAT will prove complex. For these reasons, studies investigating mechanisms leading to macrophage accumulation may yield more promising therapeutic targets.

## Potential Mechanisms for Macrophage Accumulation in Obesity

White adipose tissue macrophage accumulation is thought to occur through two main processes. First, increased chemokine secretion from adipocytes and resident macrophages promotes the recruitment of Ly6C^+^ blood monocytes to obese WAT. Most of these monocytes subsequently differentiate into M1 macrophages in response to inflammatory signals within the adipose tissue. Of the chemokines produced from obese WAT, monocyte chemoattractant protein-1(MCP-1) and its receptor C–C motif receptor-2 (CCR2) appear particularly important. For example, mice lacking either CCR2 or MCP-1 have reduced ATMs, whereas adipocyte specific over-expression of MCP-1 leads to enhanced ATMs (20, 21). However, these knockout models did not normalize macrophage numbers suggesting additional mechanisms are likely to be involved in obesity-induced macrophage accumulation (20). Indeed, obese WAT secretes many chemokines including LTB4, MIP, MIF, and MCP-3 implicated in macrophage accumulation and glucose intolerance (22). Interestingly, in addition to chemokine secretion, signals from obese WAT also influence bone marrow progenitor cells to increase myelopoiesis. Indeed, we have recently demonstrated that in obese mice, IL1β production from CD11c^+^ ATMs promoted bone marrow myelopoiesis further perpetuating adipose tissue inflammation (23). Second, resident ATMs have a strong proliferation capacity in both human beings and rodents. Indeed, Jenkins et al. showed that IL4 is a strong promoter of macrophage proliferation (24). Hence, in the lean state where eosinophils secrete high levels of IL4 in the WAT, proliferation is considered the major mechanism to maintain resident M2 macrophage populations (24–26). Interestingly, Amano et al. revealed that MCP-1 could enhance macrophage proliferation in the visceral WAT independent of its chemokine function (27). It is clear that further studies are required to determine the contribution of macrophage proliferation and recruitment to adipose tissue macrophage accumulation in obesity.

## Macrophage Fate

While the recruitment and source of macrophages present in obese adipose tissue are well documented, the fate of these recruited macrophages remains less studied. Unlike resolving inflammation in which levels of inflammatory leukocytes subside following the restoration of tissue homeostasis, adipose tissue inflammation induced by excessive adiposity fails to resolve naturally. During resolving inflammation, it is well appreciated that the initially recruited polymorphonuclear neutrophils undergo swift apoptosis prompting their phagocytosis by macrophages. Despite early evidence for macrophage emigration and drainage to lymph nodes as a significant contributor to macrophage disappearance following acute inflammation, recent work from the Randolph laboratory has revealed that macrophage apoptosis is largely responsible for their removal in acute inflammation (28). Whether these mechanisms contribute to adipose tissue macrophage accumulation in non-resolving inflammation is not well studied. For example, whether M1 macrophages accumulate within obese adipose tissue due to pro-survival/anti-apoptotic signals remains unknown. However, it is also possible that macrophages are actively retained within the adipose tissue in response to various cues. Netrin-1, best described for its role in neural development, has recently been implicated in macrophage retention within obese adipose tissue (29). However, these findings are somewhat at odds with the plethora of studies showing the importance of monocyte/macrophage recruitment, repopulation (e.g., after bone marrow transplantation), and proliferation to the macrophage burden in the obese adipose tissue and perhaps plays only a minor role.

## A More Complex Picture than M1 or M2

Macrophages are able to modify their phenotype according to their environment. However, whether macrophage phenotype switching occurs in obesity remains unresolved (30, 31). Shaul et al. demonstrated that in HFD-fed animals’ classical M1 macrophage accumulation is observed after 8 weeks of diet, however, after 12 weeks of diet, these CD11c^+^ macrophages exhibited an increased expression of M2 associated transcripts (32). While the M1 macrophages maintained their pro-inflammatory phenotype, they also adopted some remodeling features in a context of increased adipogenesis reminiscent of M2. Conversely, M2 macrophages are able to secrete pro-inflammatory mediators in specific conditions (33). These studies reveal extremely dynamic macrophage populations and newer technologies such as live imaging undoubtedly will enhance our understanding of the changes occurring in macrophage polarization state upon weight gain. Indeed, Haase et al. showed recently that most adipose tissue macrophages arising *in situ* stained positive for the M2 markers CD206 and CD301 (34). Live imagery and tracking techniques allowed the revelation that newly formed M2 macrophages originates within the CLS before they migrate to the interstitial space. Given that CLS form around dying adipocytes, the presence of M2 macrophages could be viewed as a resolving mechanism. Given that macrophages are able to alter their phenotype and that most studies assess adipose tissue macrophage content at one single time, care must be exercised when interpreting data. For example, one cannot expect to predict a metabolic phenotype based upon observed adipose tissue macrophage polarity.

It remains plausible that the inflammation associated with obesity initially constitutes a physiological rather than pathological process within the adipose tissue. Indeed, in an elegant study, Scherer and colleagues have demonstrated that adipocyte inflammation is essential for adipose tissue expansion and remodeling (35). Using the “adipochaser mouse,” they tracked newly formed adipocytes and distinguished them from older “blue” adipocytes expressing β-galactosidase. They determined that acute inflammation promotes adipogenesis and improved adipose tissue function and insulin sensitivity. Conversely, they demonstrated that abrogation of inflammation within the adipose tissue led to defective adipogenesis followed by ectopic lipid accumulation and glucose intolerance (35). These findings highlight a previously unappreciated role for inflammation *per se* in healthy adipose tissue function.

## Can We Target Immunity to Treat T2D?

It is important to ensure that the findings made in rodent models are useful in human pathology. It is clear that macrophages also accumulate in adipose tissue of obese humans (7) and have been correlated with insulin resistance (36).Wentworth et al. also showed that pro-inflammatory CD11c^+^ macrophages are positively associated with systemic insulin resistance in obese patients (37). Furthermore, macrophage content is reduced in the adipose tissue following gastric bypass surgery (38). Importantly, in these patients, the macrophage status was switched toward a less pro-inflammatory profile. However, other groups have shown that the accrued macrophages in human adipose tissue present M2 surface markers associated with a more anti-inflammatory phenotype (39, 40). Nonetheless, Zeyda et al. showed that these M2 macrophages possess a strong capacity to produce pro-inflammatory mediators (39). These discrepancies may be explained by different experimental protocols. In most studies, WAT is obtained from subcutaneous depots whereas fewer studies report data obtained from omental WAT. Furthermore, the sex and degree of adiposity of the patients may also account for some of the differences reported. The same reasons may partly explain the inconsistencies observed between mice and human beings. In addition, important metabolic differences between rodents and human beings contribute to the difficulty of translating mouse data into human therapies.

In addition, there are further evidences that inflammation and macrophages should be targeted with the greatest care in T2D. Indeed, Chawla’s group recently reported that M2 macrophages can secrete catecholamines in response to cold exposure, activating thermogenesis of brown adipose tissue and lipolysis of WAT (41). They later found that M2 macrophages were directly involved in the “beginning” of adipose tissue (42). In addition, a current article proposed that M2 macrophages strongly promote β-cell proliferation (43). The presented studies tend to suggest that the positive effects of macrophages in obesity and T2D are carried on by repair macrophages exhibiting an M2 phenotype. Hence, there appears to be rationale targeting the enhancement of M2 populations as opposed to depleting M1 macrophages, which may jeopardize the patient’s immune function. However, increased M2 macrophage function is also tightly linked to tumor proliferation so manipulating M2 populations must be considered carefully as obese patients already present with an increased incidence of cancer (2).

## Conclusion

It is increasingly appreciated that immune cells play a crucial role in the control of whole body metabolism (2). In light of this view, it seems clear that the macrophage accumulation within the adipose tissue is associated with metabolic dysfunction observed in obesity. However, the role of macrophages in the pathogenesis of obesity and diabetes is still conflicting. In addition to the complexity of the immune response itself, different rodent models, the use of different high-fat diets and of different intervention time courses not to add the different environments of each animal facility, have often led to contradictory results raising more confusion within the field. As it was nicely exposed in a recent review by Murray et al., it is important for the coherence of results in this domain that researchers try and decide which models to focus on to allow the field to progress and result in more human translational potential (5).

**Figure 1:**
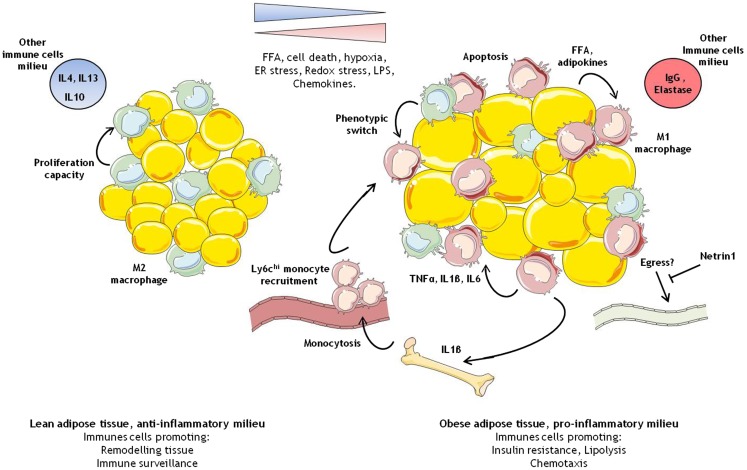
**Immune cells modulation in adipose tissue during obesity**. In the lean healthy adipose tissue, M2 like macrophages self-maintain through proliferation. They ensure tissue remodeling and pathogen screening. In the obese adipose tissue, overnutrition leads to larger adipocytes, which coupled with various intrinsic and extrinsic cellular stress, and promotes the development of a pro-inflammatory environment. In this context, M1 like macrophages start to accumulate due to some M2 macrophages switching phenotype, bone marrow stimulation of myelopoiesis contributing to the recruitment of pro-inflammatory monocytes, and possibly reduced egress of macrophages.

In this review, we have summarized the recent findings on the role of macrophages in obesity and T2D. M1 macrophage accumulation within the adipose tissue remains tightly associated with obesity. It is still unclear how much this accumulation contributes to the glucose intolerance and insulin resistance described in obese rodent models. There is growing literature suggesting their presence could be required for physiological adaptations of the adipose tissue (35). Perhaps these findings reflect the dynamic nature of macrophage polarity and the essential role of macrophages in the biology of adipose tissue (Figure 1). However, it is also important to take a “non-glucose/insulin resistant-centric view” with respect to the role of adipose tissue macrophages and to appreciate that they could also contribute significantly to the risk of other associated diseases including cardiovascular disease and cancer. As a whole, these data advocate that macrophages should be targeted with the greatest care in metabolic diseases.

## Conflict of Interest Statement

The authors declare that the research was conducted in the absence of any commercial or financial relationships that could be construed as a potential conflict of interest.
